# PPARγ silencing enhances osteogenic differentiation of human adipose-derived mesenchymal stem cells

**DOI:** 10.1111/jcmm.12098

**Published:** 2013-08-13

**Authors:** Mon-Juan Lee, Hui-Ting Chen, Mei-Ling Ho, Chung-Hwan Chen, Shu-Chun Chuang, Sung-Cheng Huang, Yin-Chih Fu, Gwo-Jaw Wang, Lin Kang, Je-Ken Chang

**Affiliations:** a Orthopaedic Research Center, Kaohsiung Medical UniversityKaohsiung, Taiwan; b Department of Bioscience Technology, Chang Jung Christian UniversityTainan, Taiwan; c Department of Fragrance and Cosmetic Science, Kaohsiung Medical UniversityKaohsiung, Taiwan; d Department of Orthopaedics, Kaohsiung Medical UniversityKaohsiung, Taiwan; e Department of Physiology, Kaohsiung Medical UniversityKaohsiung, Taiwan; f Graduate Institute of Physiology and Molecular Medicine College of Medicine, Kaohsiung Medical UniversityKaohsiung, Taiwan; g Graduate Institute of Medicine, College of Medicine Kaohsiung Medical UniversityKaohsiung, Taiwan; h Department of Sports Medicine Faculty of Medicine, Kaohsiung Medical UniversityKaohsiung, Taiwan; i Department of Orthopaedics Kaohsiung Medical University Hospital, Kaohsiung Medical UniversityKaohsiung, Taiwan; j Department of Orthopaedics, National Cheng Kung University Medical College and HospitalTainan, Taiwan; k Department of Orthopaedic Surgery, University of VirginiaVirginia, USA; l Department of Obstetrics and Gynaecology, National Cheng Kung University Medical College and HospitalTainan, Taiwan; m Department of Orthopaedics Kaohsiung Municipal Ta-Tung Hospital, Kaohsiung Medical UniversityKaohsiung, Taiwan

**Keywords:** Human adipose tissue-derived mesenchymal stem cells, peroxisome proliferator-activated receptor gamma, small interfering RNA, osteogenesis, adipogenesis

## Abstract

Peroxisome proliferator-activated receptor gamma (PPARγ) is the master regulator of adipogenesis, and has been indicated as a potential therapeutic target to promote osteoblast differentiation. However, recent studies suggest that suppression of PPARγ inhibits adipogenesis, but does not promote osteogenic differentiation in human bone marrow-derived mesenchymal stem cells (hBMSCs). It was reasoned that the osteogenic effect of PPARγ suppression may be masked by the strong osteogenesis-inducing condition commonly used, resulting in a high degree of matrix mineralization in both control and experimental groups. This study investigates the role of PPARγ in the lineage commitment of human adipose-derived mesenchymal stem cells (hADSCs) by interfering with the function of PPARγ mRNA through small interfering RNAs (siRNAs) specific for PPARγ2. By applying an osteogenic induction condition less potent than that used conventionally, we found that PPARγ silencing led to retardation of adipogenesis and stimulated a higher level of matrix mineralization. The mRNA level of PPARγ decreased to 47% of control 2 days after treatment with 50 nmol/l PPARγ2 siRNA, while its protein expression was 60% of mock control. In the meantime, osteogenic marker genes, including bone morphogenic protein 2 (BMP2), runt-related transcription factor 2 (Runx2), alkaline phosphatase (ALP) and osteocalcin (OC), were up-regulated under PPARγ silencing. Our results suggest that transient suppression of PPARγ promotes the onset of osteogenesis, and may be considered a new strategy to stimulate bone formation in bone tissue engineering using hADSCs.

## Introduction

Peroxisome proliferator-activated receptor gamma (PPARγ) is the master regulator of adipogenesis, and has been indicated as a critical switch in the commitment of progenitor cells to either the adipogenic or osteogenic pathway [1–3]. Down-regulation of PPARγ is considered a novel strategy to promote bone regeneration, which is critical in the treatment of bone defects caused by trauma or bone resorption [4]. Transient suppression of PPARγ in mouse embryonic stem (ES) cells by PPARγ–specific siRNAs directs ES cells to differentiate into an osteoblastic lineage [5]. Peroxisome proliferator-activated receptor gamma siRNAs have also been reported to enhance bone formation in human preadipocytes and foetal-femur-derived mesenchymal cells, and reverse osteogenic repression of alcohol on human bone marrow-derived mesenchymal stem cells (hBMSCs) [6, 7]. *In vivo* studies also demonstrate that mice with impaired expression of PPARγ exhibit increased bone mass [8, 9].

Recently, it was reported that suppression of PPARγ through either PPARγ antagonists or RNA interference inhibits adipogenesis, but does not promote osteogenic differentiation of hBMSCs, suggesting that PPARγ may not be the master regulator of lineage determination in human bone marrow [10]. In this study, we provide a closely related but controversial observation in human adipose-derived mesenchymal stem cells (hADSCs), in which PPARγ silencing resulted in higher level of osteogenic gene expression and matrix mineralization. The discrepancy between our study and the previous one was discussed.

## Materials and methods

### Isolation and cell culture conditions of hADSCs

The protocol for this study was approved by the institutional review board of Kaohsiung Medical University Hospital. The detailed procedures of the isolation and characterization of hADSCs have been reported previously [11, 12]. Human ADSCs were maintained in K-NAC medium, which consists of Keratinocyte-SFM (Gibco-BRL, Grand Island, NY, USA) supplemented with 2 mmol/l *N*-acetyl-L-cysteine (NAC) and 0.2 mmol/l L-ascorbate 2-phosphate (Asc 2-P) [13]. Cells were seeded at 10^6^ cells per 10-cm dish for protein extraction and western blot analysis, and at 5 × 10^5^ cells per well in 6-well plates for RNA extraction in preparation for reverse transcription polymerase chain reaction (RT-PCR) or real-time PCR. For alizarin red S or oil red O staining, hADSCs were seeded at a density of 10^5^ cells per well in 12-well plates.

### siRNA transfection

*Silencer*® Select Validated siRNA specific for the human PPARγ2 gene (5′-AAGAAATGACCATGGTTGACACAGAGAT-3′) was designed and synthesized by Life Technologies, Carlsbad, CA, USA. *Silencer*® Select Negative Control #1 siRNA (Life Technologies) or non-specific FITC-oligonucleotides (Life Technologies) were applied as negative controls to assess transfection efficiency. Transfection of siRNA was carried out 24 hrs after hADSCs were plated, using Lipofectamine RNAiMAX transfection reagent (Life Technologies) by following the manufacturer's instructions. The final siRNA concentration was 50 nmol/l.

### Reverse transcription polymerase chain reaction

Total RNA was isolated from hADSCs by the Trizol reagent (Invitrogen), and reverse transcription was carried out using the Advantage RT-for-PCR Kit (Clontech, Mountain View, CA, USA) [14, 15]. PCR was performed using Taq DNA polymerase (Yeastern Biotech, Taipei, Taiwan) with the following conditions: incubation at 94°C for 5 min., followed by 35 cycles of denaturation at 94°C for 30 sec., annealing at 55°C for 30 sec. and extension at 72°C for 30 sec. RT-PCR primer sequences were as follows: PPARγ (sense, 5′-ACTCTGGGAGATTCTCCTATT-3′; antisense, 5′-CTCCATAGTGAAATCCAGAAG-3′) and 18S rRNA (sense, 5′-CCGCAGCTAGGAATAATGGAATAGGAC-3′; antisense, 5′-ACGACGGTATCTGATCGTCTTCG-3′). The PCR product was analysed on a 1.4% agarose gel, and the level of gene expression was normalized to that of 18S rRNA.

### Real-time PCR

The mRNA level of PPARγ, bone morphogenic protein 2 (BMP2), runt-related transcription factor 2 (Runx2), alkaline phosphatase (ALP) and osteocalcin (OC) were quantified as described previously [16] using the following PCR primer pairs: PPARγ (sense, 5′-CATAAAGTCCTTCCCGCTGA-3′; antisense, 5′-GGGCTCCATAAAGTCACCAA-3′), BMP2 (sense, 5′-GGAATGACTGGATTGTGGCT-3′; antisense, 5′-TGAGTTCTGTCGGGACACAG-3′), Runx2 (sense, 5′-AGATGGGACTGTGGTTACTG-3′; antisense, 5′-GTAGCTACTTGGGGAGGATT-3′), ALP (sense, 5′-TGTAAGGACATCGCCTAC-3′; antisense, 5′-GGGAGTGCTTGTATCTCG-3′), OC (sense, 5′-CTGCAGAGTCCAGCAAAGGT-3′; antisense, 5′-CGATAGGCCTCCTGAAAGC-3′) and GAPDH (sense, 5′- GAAGGTGAAGGTCGGAGTC-3′; antisense, 5′-GAAGATGGTGATGGGATTTC -3′).

### Western blot analysis

Cell lysates obtained from hADSCs were separated by SDS-PAGE and transferred to polyvinylidene difluoride (PVDF) membrane. After blocking with 1% BSA in PBS-Triton X-100 (PBST) for 1 hr at room temperature, the membrane was washed with PBST and reacted overnight with mouse anti-human PPARγ antibody (sc-7273, Santa Cruz, Dallas, TX, USA) or mouse anti-human β-actin antibody (sc-47778; Santa Cruz). After washing with PBST, the membrane was incubated with horseradish peroxidase (HRP)-conjugated goat antimouse secondary antibody (AP-124P, Millipore, Billerica, MA, USA) for 1 hr at room temperature. Bound antibodies were visualized with Amersham ECL Western Blotting Detection Reagents (GE Healthcare Life Sciences, Uppsala, Sweden).

### Osteogenic differentiation

The concentrations of osteogenic inducing agents in the osteogenic induction medium prepared in this study were half of those in conventional ones. After treatment with PPARγ2 siRNA for 48 hrs, hADSCs were cultured in osteogenic induction medium consisting of DMEM supplemented with 5 mmol/l β-glycerol phosphate, 50 nmol/l dexamethasone and 25 μmol/l L-ascorbate 2-phosphate, for another 2 weeks. Cells were fixed with 4% paraformaldehyde and stained with alizarin red S as described in our previous studies [16].

### Adipogenic differentiation

After treatment with PPARγ2 siRNA for 48 hrs, hADSCs were cultured in adipogenic induction medium consisting of DMEM supplemented with 1 μmol/l dexamethasone, 0.5 mmol/l methyl-isobutylxanthine, 10 μg/ml insulin and 100 μmol/l indomethacin for another 10 or 14 days (corresponding to 12 or 16 days after siRNA transfection). Cells were fixed with 4% paraformaldehyde at room temperature for 10 min., and incubated in oil red O staining solution (0.36% in 60% isopropanol) for 50 min. at room temperature. Cell-bound oil red O was extracted by adding 500 μl of isopropanol per well [17]. The amount of oil red O released was determined using a spectrophotometer at a wavelength of 540 nm.

### Statistical analysis

Statistical analysis was performed on data from at least three independent experiments. Significant difference relative to the control was tested using one-way anova. Difference in the mean was determined using Duncan's new multiple range test. Levels of significance of *P* < 0.05 and 0.01 were accepted as significant and highly significant respectively.

## Results

### Suppression of PPARγ expression and adipogenic differentiation by PPARγ2 siRNA

**Fig. 1:**
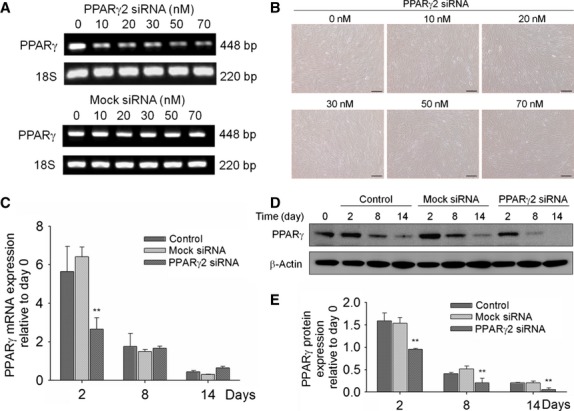
Effect of PPARγ2 siRNA on PPARγ expression and morphology of hADSCs. (**A**) Human ADSCs were treated with 0–70 nmol/l of either PPARγ2 siRNA or a non-specific oligonucleotide (mock siRNA) for 48 hrs. The mRNA level of PPARγ was determined by RT-PCR, with 18S rRNA served as the housekeeping gene. (**B**) The morphology of hADSCs treated with PPARγ2 siRNA was assessed by optical microscope under the bright field. Bar, 100 μm. (**C**) Human ADSCs were treated with 50 nmol/l PPARγ2 siRNA for 48 hrs, and cultured in osteogenic induction medium for another 0, 6 or 12 days (corresponding to 2, 8 or 14 days after siRNA transfection). The change in the mRNA level of PPARγ with time was determined by RT-PCR. (**D**) PPARγ protein expression was determined 2, 8 or 14 days after siRNA transfection by western blot analysis. (**E**) Protein levels in (**D**) was quantified by densitometry, normalized to those of β-actin, and expressed as folds relative to day 0 (*n* = 3, **P < 0.01 compared with control and mock).

**Fig. 2:**
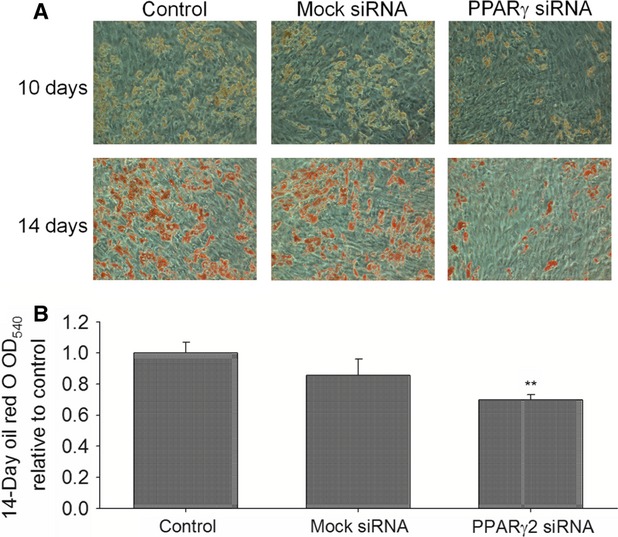
Effect of PPARγ suppression on adipogenic differentiation of hADSCs. Human ADSCs were transfected with 50 nmol/l PPARγ2 siRNA for 48 hrs, and cultured in adipogenic induction medium for another 10 or 14 days (corresponding to 12 or 16 days after siRNA transfection). (**A**) Lipid accumulation was determined by oil red O staining. (**B**) The amount of cell-bound oil red O was quantified spectrophotometrically at 540 nm (*n* = 3, **P < 0.01 compared with control and mock).

Human ADSCs were transiently transfected with either PPARγ2-specific siRNA or a non-specific control oligonucleotide (mock siRNA), and PPARγ gene expression was analysed by RT-PCR at various PPARγ2 siRNA concentrations (10–70 nmol/l). Two days after siRNA transfection, the mRNA level of PPARγ was lowered in a dose-dependent manner in the presence of 10–70 nmol/l PPARγ2 siRNA (Fig. 1A, upper panel), whereas mock siRNA had no effect on the expression of PPARγ (Fig. 1A, lower panel). In addition, cell morphology of hADSCs was not affected by PPARγ2 siRNA within the concentration range tested (Fig. 1B). As determined by real-time PCR, treatment of hADSCs with 50 nmol/l PPARγ2 siRNA for 2 days resulted in 53–59% suppression of PPARγ gene expression, compared with cells without siRNA treatment or those treated with mock control (Fig. 1C). PPARγ protein expression decreased with time in both control and siRNA-treated hADSCs, because of incubation in osteogenic induction medium after siRNA treatment. Nevertheless, treatment with PPARγ2 siRNA resulted in further reduction in intracellular PPARγ level compared to the controls (Fig. 1D). In PPARγ2 siRNA-treated cells, the amount of PPARγ protein was ∼60% of the controls 2 days after transfection (Fig. 1E). When hADSCs were treated with PPARγ2 siRNA followed by adipogenic induction for 2 weeks, lipid accumulation was significantly reduced compared to the control, indicating that adipogenic differentiation was blocked by interfering with the function of PPARγ (Fig. 2).

### Effect of PPARγ suppression on osteogenic differentiation

**Fig. 3:**
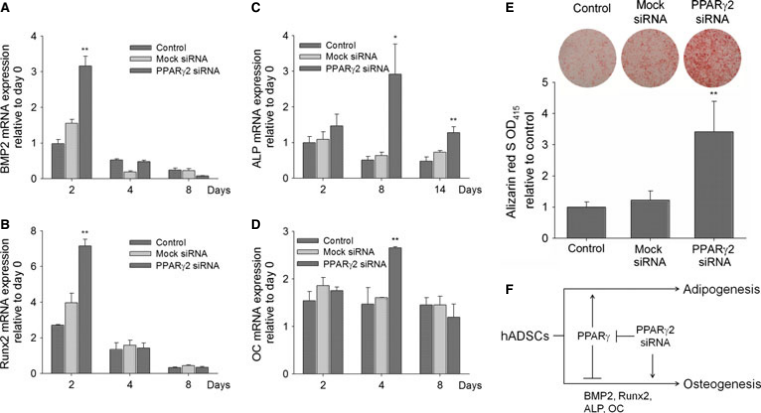
Effect of PPARγ suppression on osteogenic differentiation of hADSCs. Human ADSCs were transfected with 50 nmol/l PPARγ2 siRNA for 48 hrs, and cultured in osteogenic induction medium for another 0, 2, 6 or 12 days (corresponding to 2, 4, 8 or 14 days after siRNA transfection) to determine the mRNA level of (**A**) BMP2, (**B**) Runx2, (**C**) ALP and (**D**) OC. (**E**) Cells were induced to differentiate osteogenically as described in Materials and methods. The level of matrix mineralization was determined by alizarin red S staining 14 days after siRNA transfection (*n* = 3, **P < 0.01 compared with control and mock). (**F**) The role of PPARγ in lineage differentiation of hADSCs and the effect of PPARγ suppression as concluded from this study.

Under PPARγ suppression, the mRNA level of BMP2 and Runx2 was significantly higher than the controls 2 days after siRNA transfection (Fig. 3A and B), whereas ALP was significantly up-regulated at days 8 and 14 (Fig. 3C). The mRNA expression of OC, one of the late-onset genes associated with osteogenesis, was significantly higher than the controls 4 days after transfection of PPARγ2 siRNA (Fig. 3D). When conventional osteogenic inducing conditions (DMEM supplemented with 10 mmol/l β-glycerol phosphate, 100 nmol/l dexamethasone and 50 μmol/l L-ascorbate 2-phosphate) were applied, no significant difference in matrix mineralization was observed between hADSCs treated with PPARγ2 siRNA and the control (data not shown). However, when hADSCs were induced to differentiate under a relative mild condition (DMEM supplemented with 5 mmol/l β-glycerol phosphate, 50 nmol/l dexamethasone and 25 μmol/l L-ascorbate 2-phosphate), hADSCs transfected with PPARγ2 siRNA had a significantly higher level of calcium deposition than control cells (Fig. 3E). These results indicate that osteogenic differentiation of hADSCs was accelerated by PPARγ silencing, which may promote terminal differentiation of hADSCs towards the osteoblast lineage by up-regulating the expression of osteogenic genes.

## Discussion

The signalling cascades related to PPARγ are potential therapeutic targets to promote osteoblast differentiation in osteoporosis and age-related osteopenia and therapeutic methods that act through suppression of PPARγ or inhibition of its ligand synthesis have been proposed [3, 18]. However, recent studies indicate that suppression of PPARγ does not promote osteogenic differentiation of hBMSCs [10], which is in contrast to the results of this and several other studies [5–7]. We reasoned that in *in vitro* studies, the osteogenic effect of PPARγ suppression may be masked by the strong osteogenesis-inducing condition as used in the literature, resulting in a high degree of matrix mineralization in both control and experimental groups [10]. When a less potent induction condition was applied, the difference in the level of matrix mineralization between PPARγ-silenced and control cells can be discerned (Fig. 3E). It is also possible that there exist a difference in sensitivity to PPARγ suppression between hADSCs used in this study and hBMSCs reported in the literature [10]. Further genome-wide gene expression profiling on stem cells and precursor cells of various stages of maturation or animal models with PPARγ silencing [19] is necessary to elucidate the role of PPARγ in determining the differentiation fate.

In this study, we demonstrated that transient suppression of PPARγ prior to osteogenic induction resulted in up-regulation of osteogenic genes such as BMP2, Runx2, ALP and OC (Fig. 3A–D), and a higher level of matrix mineralization (Fig. 3E). Our results suggest that PPARγ silencing may promote the activation of BMP2 and Runx2/Cbfa1, as well as the downstream transcription of ALP and OC to induce the onset of osteogenic differentiation in hADSCs (Fig. 3F). In conclusion, our study indicates that osteogenesis of hADSCs can be enhanced by suppressing adipogenesis through PPARγ silencing. To the best of our knowledge, this is the first report to demonstrate that transient suppression of PPARγ prior to osteogenic induction is sufficient to stimulate differentiation of hADSCs towards the osteoblast lineage. This investigation highlights possible therapeutic interventions targeting PPARγ-related pathways to promote skeletal tissue regeneration.
